# Pancreaticoduodenectomy after postoperative gastric tube reconstruction for esophageal cancer with median arcuate ligament syndrome: a case report

**DOI:** 10.1186/s40792-024-01974-y

**Published:** 2024-07-29

**Authors:** Hideki Izumi, Hisamichi Yoshii, Rika Fujino, Shigeya Takeo, Yukiko Kojima, Junichi Kaneko, Masaya Mukai, Osamu Chino, Hiroyasu Makuuchi

**Affiliations:** 1 Department of Gastrointestinal Surgery, Tokai University Hachioji Hospital, 1838 Ishikawa, Hachioji, Tokyo 192-0032 Japan; 2 Department of Gastrointestinal Surgery, Tokai University Tokyo Hospital, 1-2-5 Yoyogi, Shibuya, Tokyo 151-0053 Japan

**Keywords:** Pancreaticoduodenectomy, Median arcuate ligament syndrome, Gastric tube reconstruction, Pancreatic head arcade, Esophageal cancer

## Abstract

**Background:**

Pancreaticoduodenectomy (PD) is considered a challenging surgery for resecting the gastroduodenal artery (GDA), right gastric artery (RGA), and lymph node tumors. In cases of pancreatic head cancer surgery, vascular anastomosis or right gastroepiploic artery (RGEA)/GDA preservation is necessary after postoperative gastric tube reconstruction for esophageal cancer. Therefore, we report for the first time an extremely rare case of PD in a patient with pancreatic head cancer and median arcuate ligament syndrome (MALS) after gastric tube reconstruction following esophageal cancer surgery, in which the entire pancreatic head arcade was preserved.

**Case presentation:**

The patient was a 76-year-old man who had undergone esophageal cancer surgery after sternal gastric tube reconstruction 7 years ago. He was referred to our hospital because of the suspicion of intraductal papillary mucinous carcinoma (IPMC) owing to an enlarged cystic lesion and a substantial component in the uncinate process of the pancreas. Preoperative three-dimensional computed (3D-CT) tomography angiography showed celiac axis stenosis and pancreatic head arcade dilation. The diagnosis was IPMC without evidence of invasion; therefore, gastric tube blood flow was maintained by preserving the GDA and RGEA. Due to MALS, the GDA blood flow was supplied through the pancreatic head arcade, necessitating its preservation. The GDA-RGEA, right gastroepiploic vein, and anterior superior pancreaticoduodenal artery were taped over the entire pancreatic head for preservation. The inferior pancreaticoduodenal artery (IPDA) was also taped on the dorsal pancreas and the posterior or anterior IPDA, which further bifurcates were taped to preserve them. Subsequently, PD was performed.

**Conclusion:**

We report a case of PD after gastric tube reconstruction for esophageal cancer with MALS, in which the pancreatic head arcade vessels were successfully preserved using 3D-CT to confirm the operation of the vessels.

## Background

Pancreaticoduodenectomy (PD) is considered a challenging surgery for resecting gastroduodenal artery (GDA), right gastric artery (RGA), and lymph node tumors [1, 2]. In cases of pancreatic head cancer surgery, vascular anastomosis, or right gastroepiploic artery (RGEA)/GDA preservation, is necessary after postoperative gastric tube reconstruction for esophageal cancer. Hemodynamic considerations, such as vascular reconstruction and arcade preservation, are also essential for PD with celiac artery stenosis caused by median arcuate ligament compression syndrome (MALS).

Therefore, in this study, we present the first case of PD in a patient with pancreatic head cancer with MALS after gastric tube reconstruction following esophageal cancer surgery in which the entire pancreatic head arcade was preserved.

## Case presentation

The patient was a 76-year-old Japanese man who underwent esophageal cancer surgery at another hospital 7 years ago and had a posterior sternal gastric tube reconstruction. During the esophageal cancer surgery, a 20-mm branched intraductal papillary mucinous carcinoma (IPMC) was observed in the uncinate process of the pancreas; however, it was followed up due to its small size. Subsequently, the cyst at the uncinate process of the pancreas gradually increased in size, and the patient was referred to our hospital for surgery. His medical history included a stroke but no apparent paralysis. The patient’s family history was unremarkable. There was no recurrence of esophageal cancer 7 years postoperatively. Upon admission, the patient’s blood test results were normal. The tumor markers used were carcinoembryonic and carbohydrate 19-9 antigens at 1.8 ng/mL and 40.7 U/mL, respectively.

**Fig. 1:**
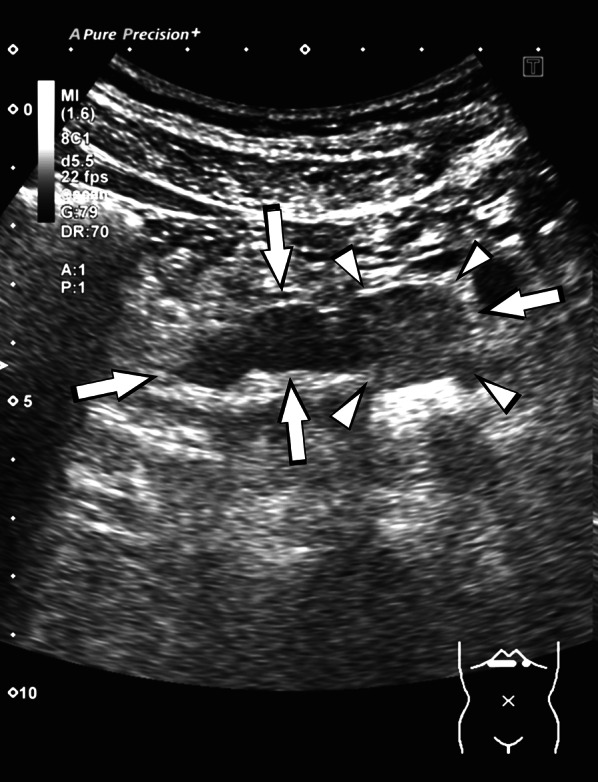
Ultrasound examination of the uncinated process of the pancreas. Rightwards double arrow shows a cystic lesion and white down-pointing triangle shows a solid component

**Fig. 2:**
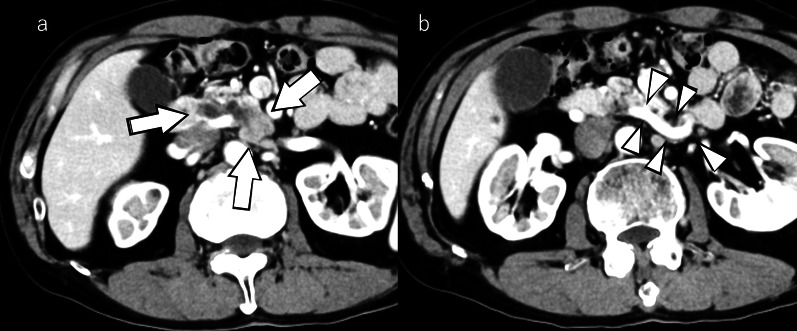
Contrast-enhanced CT of the abdomen. **a** Cystic lesion with nodules in the uncinated process of the pancreas. **b** Dilated pancreatic head arcade

**Fig. 3:**
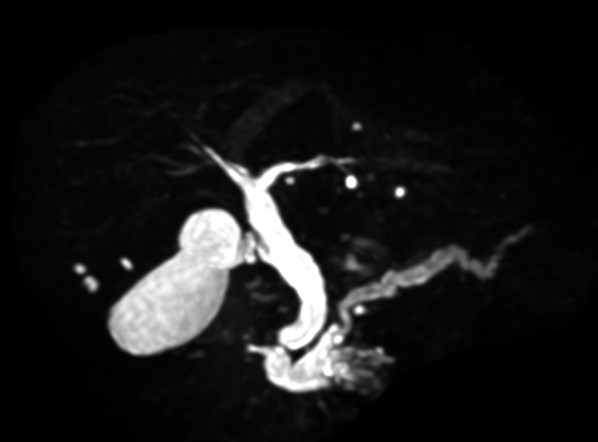
MRCP of the abdomen. The image shows a cystic lesion in the uncinated process of the pancreas and dilatation of the main pancreatic duct. *MRCP* magnetic resonance cholangiopancreatography

**Fig. 4:**
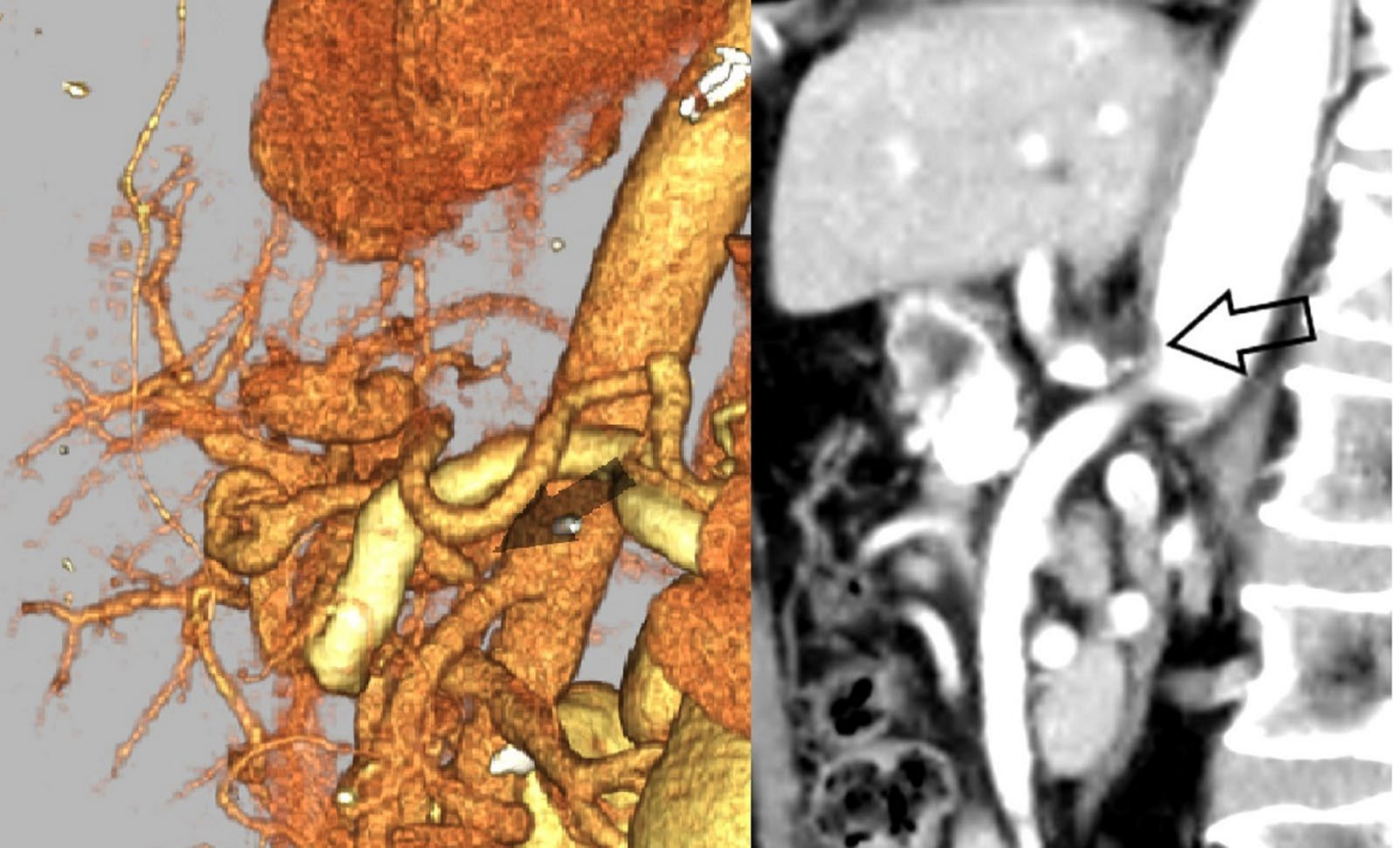
Arrow points to the MALS

**Fig. 5:**
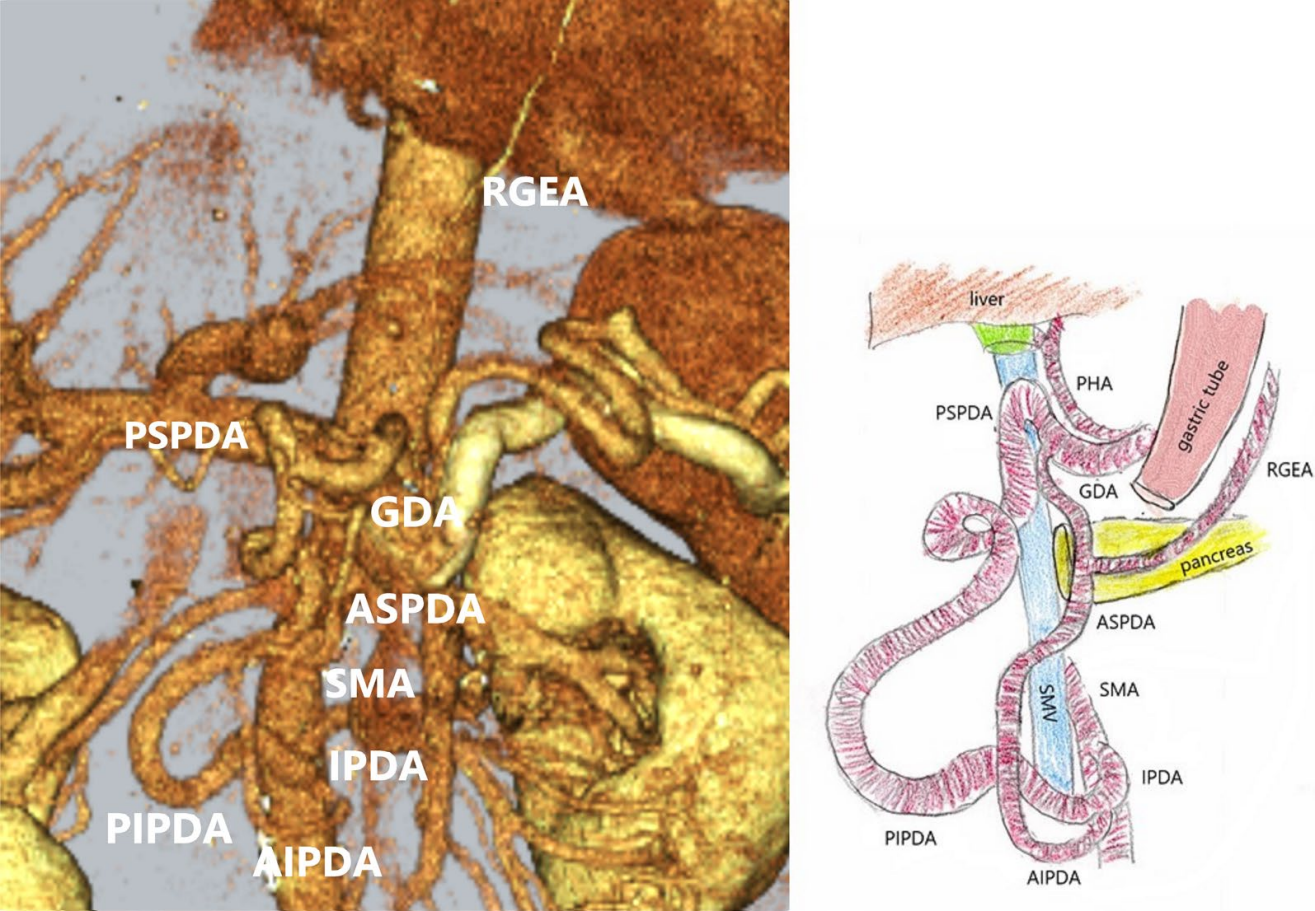
Dilated pancreatic head arcade

Abdominal ultrasonography revealed a 57-mm large cystic lesion in the uncinate process of the pancreas, with an internal nodular lesion measuring approximately 27 mm (Fig. 1). Contrast-enhanced computed tomography (CT) of the abdomen revealed a cystic lesion with nodules in the same area (Fig. 2a). However, no tumor invasion was observed in the surrounding area. The dilatation of the vessels in the pancreatic head arcade was also observed (Fig. 2b). Magnetic resonance cholangiopancreatography showed a cystic lesion in the pancreatic uncinate process and dilatation of the main pancreatic duct (Fig. 3). The three-dimensional CT (3D-CT) image revealed stenosis of the celiac axis (Fig. 4) and dilation of the pancreatic head arcade (Fig. 5). Therefore, a mixed type IPMC of the pancreas was diagnosed. Moreover, we identified abnormal blood flow in the pancreatic head arcade associated with MALS. The GDA-RGEA, RGA, and right gastroepiploic vein (RGEV) were to be preserved to protect the gastric tube after esophageal cancer surgery. From 3D-CT, it was determined that the GDA blood flow was likely to be through the superior mesenteric artery (SMA)–posterior inferior pancreaticoduodenal artery (PIPDA)–posterior superior pancreaticoduodenal artery (PSPDA)-GDA and that the pancreatic head arcade must be preserved to preserve the GDA-RGEA blood flow. The patient did not present with clinical MALS symptoms; therefore, it was determined that a ligamentectomy was unnecessary if the pancreatic head arcade could be preserved.

**Fig. 6:**
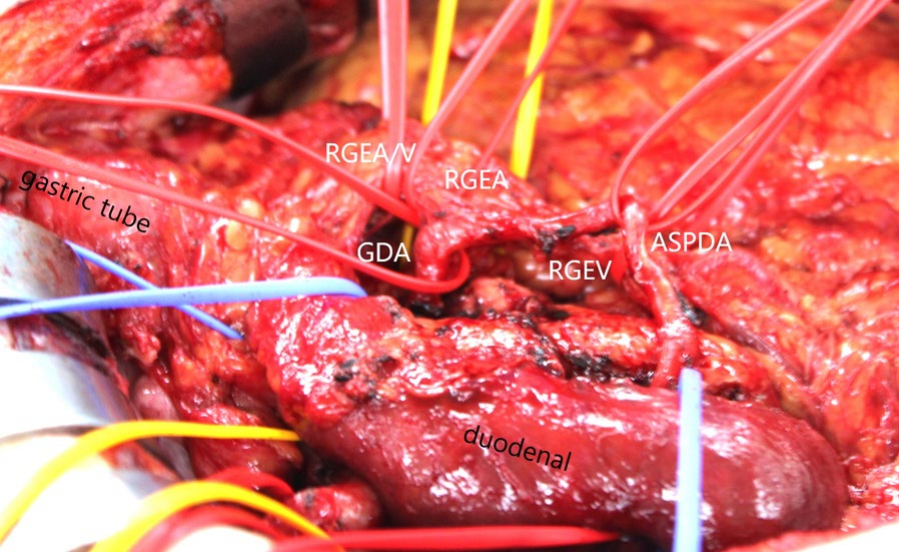
Taped and preserved GDA, RGEA, RGEV, and ASPDA. *GDA* gastroduodenal artery, *RGEA* right gastroepiploic artery, *RGEV* right gastroepiploic vein, *ASPDA* anterior superior pancreaticoduodenal artery

**Fig. 7:**
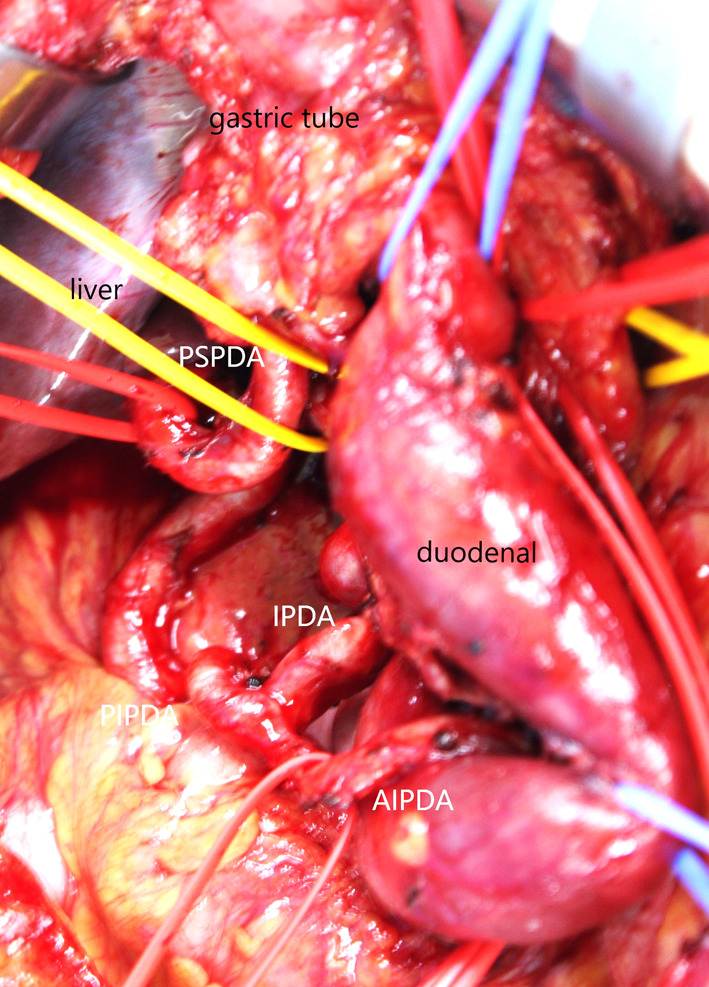
Taped and preserved PIPDA–PSPDA. *PIPDA–PSPDA* posterior inferior pancreaticoduodenal artery–posterior superior pancreaticoduodenal artery

A laparotomy was performed through a median, upper abdominal incision. Ascites cytology was performed and confirmed negative. Furthermore, peritoneal dissemination or liver metastasis was not observed. The GDA, RGEA, RGEV, and anterior superior pancreaticoduodenal artery were taped and preserved (Fig. 6). Kocher’s maneuver was performed to mobilize the duodenum, and the dorsally dilated PIPDA–PSPDA was taped and preserved (Fig. 7). The Treitz ligament was opened, and the jejunum was dissected. Subsequently, cholecystectomy and bile duct resection were performed, and the duodenum was resected. The pancreas was resected just above the portal vein, and the tumor was removed. The pancreatectomy margins were negative; an indocyanine green fluorescence imaging system (ICG-FS) confirmed that blood flow to the gastric tube was unaffected, and reconstruction was performed using the modified child’s method. The pancreatic duct was anastomosed to the jejunal mucous membrane using the modified Blumgart method, and no stents were placed in pancreatic ducts. Bile duct jejunostomy was performed on the posterior wall nodule and the continuous anterior wall. Duodenal jejunostomy was performed using the Albert–Lembert anastomosis from the end to the side. A closed suction drain was inserted posteriorly to the anastomosis between the pancreatic duct and jejunum. The surgery was completed without gastric tube complications. The surgery lasted 342 min, and the blood loss was 426 mL.

Histopathological examination of the resected specimen revealed a noninvasive, gastric-type IPMC (20 × 17 × 35 mm) of the pancreas without lymph node metastases, and the surgical margins were negative.

The patient experienced no major complications postoperatively and was discharged within 10 days.

## Discussion

Stenosis or occlusion of the celiac axis occurs in 12.5–49.7% of abdominal angiograms [3, 4] and in 4–11% of PD cases [5, 6]. The occlusion of the origin of the celiac axis is rare, and it occurs in 1.6–2.0% of PD cases [5, 7]. We previously reported a case of pancreatic head cancer in which the GDA, RGEA, and RGEV were preserved after esophageal cancer surgery and gastric tube reconstruction [8]. In this case report, a patient with pancreatic head cancer after postoperative gastric tube reconstruction for esophageal cancer had an obstruction at the origin of the celiac artery. Preoperative 3D-CT showed that the blood flow in the gastroduodenal duct primarily originated from the PIPDA to the PSPDA, GDA, RGA, and RGEA/RGEV. This is an extremely rare case of pancreatic head cancer with MALS in which gastric tube blood flow was preserved after esophageal cancer surgery by preserving all pancreatic head vascular arcades.

Performing PD after postoperative reconstruction of the gastric tube following esophageal cancer surgery is extremely difficult due to the preservation of gastric tube blood flow.

We previously reported a case where complete resection could be achieved without compromising gastric tube blood flow by preserving the GDA and RGEA owing to the absence of vascular invasion [8]. Despite concerns regarding inadequate lymph node dissection, the patient is still alive and recurrence-free for > 5 years. Ikeda et al. [9] also reported PD with GDA-RGEA preservation in two cases. Inoue et al. [10] reported that after the standard PD, GDA and RGEA were anastomosed to protect gastric tube blood flow. Additionally, cases have been documented where the middle colic artery (MCA) and RGEA were reconstructed [11, 12]. In PD cases, preservation of the GDA or RGEA may not be possible after esophageal cancer surgery and gastric tube reconstruction if the cancer has invaded these vessels. When the GDA is removed, reconstruction should be performed with revascularization to preserve the gastric tube, or the gastric tube should be removed along with reconstruction using other organs. In either case, preserving the GDA to the greatest extent possible is preferable because surgical invasion is expected to be much more invasive. In this study, we preserved the artery and the RGEV. The RGEV and left renal vein [13] and the RGEV and middle colic vein [14] were reconstructed after RGEV dissection; however, no blood flow disorder or congestion was reported in the dissected cases [15, 16]. Reports regarding RGEV preservation vary and are contradictory. Preserving the RGEV to the greatest extent is preferable because of the possibility of blood flow obstruction.

Stenosis or occlusion of the celiac axis occurs in 4–11% of PD cases [5, 6]. In patients with this condition, the blood supply to the upper abdominal organs is of little clinical significance because blood flow is supplied from the SMA, mainly the IPDA, to the GDA through the pancreaticoduodenal artery [7]. In patients undergoing resection of the pancreatic head region with celiac axis stenosis, GDA dissection can cause ischemia in the liver, stomach, and remaining pancreas, increasing the risk of suture failure [17, 18]. PD has been performed severally without diagnosing MALS, which caused ischemia [19–21]. Therefore, preoperative and intraoperative assessments of blood flow are essential in PD cases. The GDA clamp test is widely used as a convenient method of intraoperative evaluation of blood flow in cases of MALS [7, 22, 23]. Nara et al. [22] recommended evaluating the GDA clamp test with Doppler echocardiography and performing revascularization if the intrahepatic blood flow is reduced. The ICG-FS is a useful device for evaluating real-time blood flow in various organs [24]. Therefore, treatment for PD is necessary in cases in which reduced blood flow is expected. In cases of MALS with extrinsic compression, the commonly performed procedure is arch ligamentotomy, which is ineffective in cases of intrinsic stenosis such as arteriosclerosis [6, 25]. Other reported methods include revascularization, stenting, and arcade preservation [26, 27]. MCA-GDA is often used for reconstruction [4, 28]; however, GDA/RGEA preservation was necessary in the present case, ruling out MCA-GDA reconstruction. Other reported methods of vascular reconstruction include splenic artery-aorta [29]. However, reconstruction is difficult, and protecting the vascular anastomosis was difficult and expected to be a serious complication in the event of a pancreatic leakage. Preoperative stenting was considered; however, the patency rate of stents placed in the celiac axis is 60–75% at 1 year postoperatively [30], necessitating restenosis. Notably, several studies have shown a low incidence of ischemic complications after typical PD without preoperative intervention or GDA reconstruction in patients with celiac artery stenosis [7, 31]. In this case, reaching the celiac axis was challenging because of the covering of the reconstructed gastric tube; therefore, we decided to preserve the pancreatic head arcade.

## Conclusions

Here, we reported a case of PD with preserved pancreatic head arcade after postoperative gastric tube reconstruction for esophageal cancer with MALS. The preoperative diagnosis was low-grade IPMC; therefore, preservation of the pancreatic head arcade may be curative. The absence of preoperative symptoms owing to MALS is a deciding factor in preserving the pancreatic head arcade. Furthermore, the possibility of causing an aneurysm of the pancreatic head arcade cannot be ruled out; therefore, strict follow-up is necessary.

## Data Availability

Not applicable.

## Abbreviations

PD: Pancreaticoduodenectomy
MALS: Median arcuate ligament compression syndrome
IPMC: Intraductal papillary mucinous carcinoma
3D-CT: 3-Dimensional computed tomography angiography
GDA: Gastroduodenal artery
RGEA: Right gastroepiploic artery
RGEV: Right gastroepiploic vein, anterior
IPDA: Inferior pancreaticoduodenal artery
PIPDA: Posterior inferior pancreaticoduodenal artery
AIPDA: Anterior inferior pancreaticoduodenal artery
RGA: Right gastric artery
CT: Computed tomography
SMA: Superior mesenteric artery
PSPDA: Posterior superior pancreaticoduodenal artery
ICG-FS: Indocyanine green fluorescence imaging system
MCA: Middle colic artery
